# Supplementary material for: Facing the Green Threat: A Water Flea’s Defenses against a Carnivorous Plant
Source: Int J Mol Sci. 2022 Jun 9;23(12):6474. doi: 10.3390/ijms23126474 (PMC9223663; doi:10.3390/ijms23126474)
Supplement: Supplementary file 1 [file ijms-23-06474-s001.zip › ijms-1753049-supplementary.pdf]

Table S1: Number of total animals measured in the 10 replicates of the 6-day experiment as well as the ANOVA F-values of the respective statistics (Fig 1). All shown ANOVAs had 3 degrees of freedom (*DF*).

<table>
<tr><th>Day</th><th colspan="3">1</th><th colspan="3">2</th><th colspan="3">3</th><th colspan="3">4</th><th colspan="3">5</th><th colspan="3">6</th></tr>
<tr><th rowspan="2">Treatment</th><th rowspan="2">n</th><th colspan="2">ANOVA F-value</th><th rowspan="2">n</th><th colspan="2">ANOVA F-value</th><th rowspan="2">n</th><th colspan="2">ANOVA F-value</th><th rowspan="2">n</th><th colspan="2">ANOVA F-value</th><th rowspan="2">n</th><th colspan="2">ANOVA F-value</th><th rowspan="2">n</th><th colspan="2">ANOVA F-value</th></tr>
<tr><th>Body length</th><th>Body width</th><th>Body length</th><th>Body width</th><th>Body length</th><th>Body width</th><th>Body length</th><th>Body width</th><th>Body length</th><th>Body width</th><th>Body length</th><th>Body width</th></tr>
<tr><td>tap water control</td><td>84</td><td rowspan="4">3.38</td><td rowspan="4">3.07</td><td>69</td><td rowspan="4">6.6</td><td rowspan="4">1.14</td><td>45</td><td rowspan="4">14.15</td><td rowspan="4">4.52</td><td>63</td><td rowspan="4">17.42</td><td rowspan="4">6.45</td><td>73</td><td rowspan="4">32.95</td><td rowspan="4">14.72</td><td>55</td><td rowspan="4">29.21</td><td rowspan="4">17.25</td></tr>
<tr><td>Ceratophyllum</td><td>37</td><td>30</td><td>35</td><td>25</td><td>44</td><td>29</td></tr>
<tr><td>fed Utricularia</td><td>79</td><td>72</td><td>42</td><td>50</td><td>50</td><td>43</td></tr>
<tr><td>unfed Utricularia</td><td>69</td><td>59</td><td>38</td><td>49</td><td>64</td><td>43</td></tr>
</table>

Table S2: Mean body length and body width in mm ±SD of *C. dubia* over the 6 days of the experiment.

**Treatment**	**Day 1**		**Day 2**		**Day 3**		**Day 4**		**Day 5**		**Day 6**	
	Body length	Body width	Body length	Body width	Body length	Body width	Body length	Body width	Body length	Body width	Body length	Body width
**tap water control**	0.49±0.03	0.30±0.02	0.59±0.05	0.36±0.04	0.66±0.05	0.41±0.04	0.71±0.07	0.49±0.07	0.74±0.06	0.52±0.05	0.77±0.05	0.56±0.05
Ceratophyllum	0.50±0.03	0.30±0.02	0.60±0.05	0.37±0.04	0.64±0.07	0.42±0.07	0.67±0.06	0.45±0.07	0.71±0.07	0.49±0.07	0.76±0.06	0.54±0.06
**fed *Utricularia***	0.48±0.03	0.29±0.02	0.55±0.07	0.33±0.05	0.57±0.09	0.35±0.08	0.62±0.07	0.41±0.07	0.63±0.08	0.42±0.08	0.67±0.06	0.46±0.06
**unfed *Utricularia***	0.50±0.05	0.30±0.03	0.57±0.07	0.36±0.05	0.60±0.06	0.38±0.04	0.65±0.06	0.44±0.06	0.67±0.05	0.45±0.05	0.71±0.05	0.49±0.05

Table S3: Bonferroni corrected pairwise t-test results for 2D morphology body length analysis. Significant results marked in red.

Day	1			2			3		
	Tap water ctrl	fed *Utric.*	*Cer.* ctrl	Tap water ctrl	fed *Utric.*	*Cer.* ctrl	Tap water ctrl	fed *Utric.*	*Cer.* ctrl
fed *Utricularia*	0.197			0.0022			4.9e-8		
Ceratophyllum ctrl	1	0.176		1	0.0013		1	4.5e-5	
unfed *Utricularia*	1	0.023	1	1	0.187	0.3159	0.0042	0.0954	0.1835

Day	4			5			6		
	Tap water ctrl	fed *Utric.*	*Cer.* ctrl	Tap water ctrl	fed *Utric.*	*Cer.* ctrl	Tap water ctrl	fed *Utric.*	*Cer.* ctrl
fed *Utricularia*	2.8e-10			<2e-16			2.6e-14		
Ceratophyllum ctrl	0.1649	0.0077		0.2034	6.8e-9		1	4.2e-9	
unfed *Utricularia*	5.3e-5	0.1474	0.9692	2.7e-8	0.0025	0.0079	5e-6	0.0066	0.0029

Table S4: Bonferroni corrected pairwise t-test results for 2D morphology body width analysis. Significant results marked in red.

Day	1			2			3		
	Tap water ctrl	fed *Utric.*	*Cer.* ctrl	Tap water ctrl	fed *Utric.*	*Cer.* ctrl	Tap water ctrl	fed *Utric.*	*Cer.* ctrl
fed *Utricularia*	0.34			1			1		
Ceratophyllum ctrl	1	0.04		1	1		0.056	0.0026	
unfed *Utricularia*	1	0.16	1	1	0.52	1	1	0.7674	0.2504
Day	4			5			6		
	Tap water ctrl	fed *Utric.*	*Cer.* ctrl	Tap water ctrl	fed *Utric.*	*Cer.* ctrl	Tap water ctrl	fed *Utric.*	*Cer.* ctrl
fed *Utricularia*	0.00024			7.9e-8			5.2e-8		
Ceratophyllum ctrl	0.06692	1		0.1841	0.0085		1	0.001	
unfed *Utricularia*	0.62437	0.09849	1	3.3e-6	1	0.1059	4.8e-7	1	0.004

Table S5: Mean number off eggs ±SD for each treatment on day 3, 4, 5 and 6.

Treatment	Day 3	Day 4	Day 5	Day 6
**tap water control**	0±1.66	2±1.51	2±1.65	2±1.86
Ceratophyllum	0±1.20	0±1.18	1±1.71	2.5±1.73
**fed *Utricularia***	0±1.11	1±1.00	0.5±1.14	1±0.68
**unfed *Utricularia***	0±1.71	2±1.45	0±1.20	1±1.13
